# Case Report: Extraction of a stylet-driven lead for left bundle branch area pacing >2 years after implantation

**DOI:** 10.3389/fcvm.2024.1457025

**Published:** 2024-08-26

**Authors:** Ivana Grgic Romic, Ana Lanca Bastiancic, David Zidan, Mate Mavric, Sandro Brusich

**Affiliations:** Department of Cardiovascular Diseases, Rijeka University Hospital Centre, Rijeka, Croatia

**Keywords:** conduction system pacing, left bundle branch area pacing, stylet-driven lead, transvenous lead extraction, venoplasty

## Abstract

Left bundle branch pacing has recently emerged as a significant alternative to right ventricular pacing. The rate of implanted stylet-driven septal leads is expected to increase substantially in the coming years, along with the need to manage long-term complications. Experience in extracting these leads is currently very limited; however, the number of complex extractions is anticipated to increase in the future. We report a complex case involving the extraction of a long-dwelling Solia lead used for left bundle branch pacing in a 21-year-old man. The lead was extracted through the implant vein 27 months after implantation, using a methodology that involved a locking stylet and compression coil. The new lead insertion was challenging due to venous occlusion but after successful venoplasty, the His lead was successfully implanted. The postoperative course was uneventful, demonstrating the feasibility of extraction without complications.

## Introduction

Transvenous lead extraction is a challenging procedure with a high risk of complications (1). The number of lead extractions has evolved over the years following the expansion of indications for device therapy (1). Conduction system pacing (CSP) is rapidly replacing right ventricular apical pacing to avoid the unfavourable effects of ventricular dyssynchrony (2, 3). Extensive studies are reviewing the clinical effectiveness of CSP, directly comparing it with cardiac resynchronisation therapy, with promising results (4–6). Consequently, the rate of implanted stylet-driven (SD) septal leads will likely increase significantly in the upcoming years (7), as will the need to manage short- and long-term complications. We describe a case of a transvenous extraction of an SD lead for left bundle branch area pacing (LBBAP), showing the feasibility of extraction in a long-term setting without complications.

## Case description

A 21-year-old man was referred to our institution for the extraction of an LBBAP SD lead. His medical history revealed that he had undergone successful minimal invasive surgical aortic valve replacement (On-X mechanical valve 27–29 mm, On-X Life Technologies, Austin, TX, USA) at the age of 19 due to severe symptomatic aortic regurgitation. Intraoperatively, complete AV block with a narrow QRS complex was noted, and on the 6th postoperative day, a permanent single-chamber pacemaker with atrial sensing (VDD) was implanted*.* Owing to issues with atrial oversensing observed during follow-up (Figure 3A), it was decided to implant a dual-chamber pacemaker with a conduction system pacing lead.

Two months after his first pacemaker implant, a delivery sheath (Selectra 3D 55-42, BIOTRONIK, SE & Co., KG, Berlin, Germany) was used to implant an SD lead (Solia S60, BIOTRONIK, SE & Co., KG) into the 12-mm thick interventricular septum in the left bundle branch area, approximately 2 cm distal to the His region, through the left axillary vein. The final parameters were a left ventricular activation time (LVAT) in V6 of 80 ms, QRS length of 110 ms, sensing of the R wave of 15 mV, pacing impedance of 830 ohms, and pacing threshold of <1 V at 1 ms.

The His position was initially targeted but there was no capture at 5 V at 1 ms at the site of the His bundle potentials. A conventional active atrial lead (Solia 53, BIOTRONIK SE & Co. KG) was implanted in the right atrial appendage also via the left axillary vein. The right ventricular lead was easily extracted from the right ventricular apex. Leads were connected to the dual-chamber pacemaker (Adapta DR, Medtronic Inc., Minneapolis, MN, USA).

In the next 2 years after the LBBAP implantation, echocardiography examinations revealed a gradual reduction in the left ventricular ejection fraction, decreasing from 51% to 35%. Upon reviewing electrocardiographic (ECG) tracings, it was observed that the initially optimal LBBAP QRS morphology began resembling deep septal pacing (Figure 3B). Follow-up pacing parameters were also less optimal, likely due to late lead microdislocation. Consequently, the decision was made to refer the patient for LBBAP lead extraction and plan redo cardiac resynchronisation therapy.

The patient came to our facility 27 months after the LBBAP implantation. The procedure was performed under general anaesthesia with a cardiothoracic surgery team on standby in the operating room. The procedure was monitored using intracardiac echocardiography, which was inserted through the right femoral vein, and fluoroscopic guidance. Preprocedural ipsilateral contrast venography revealed a complete occlusion of the left subclavian vein (Figure 2A).

**Figure 1:**
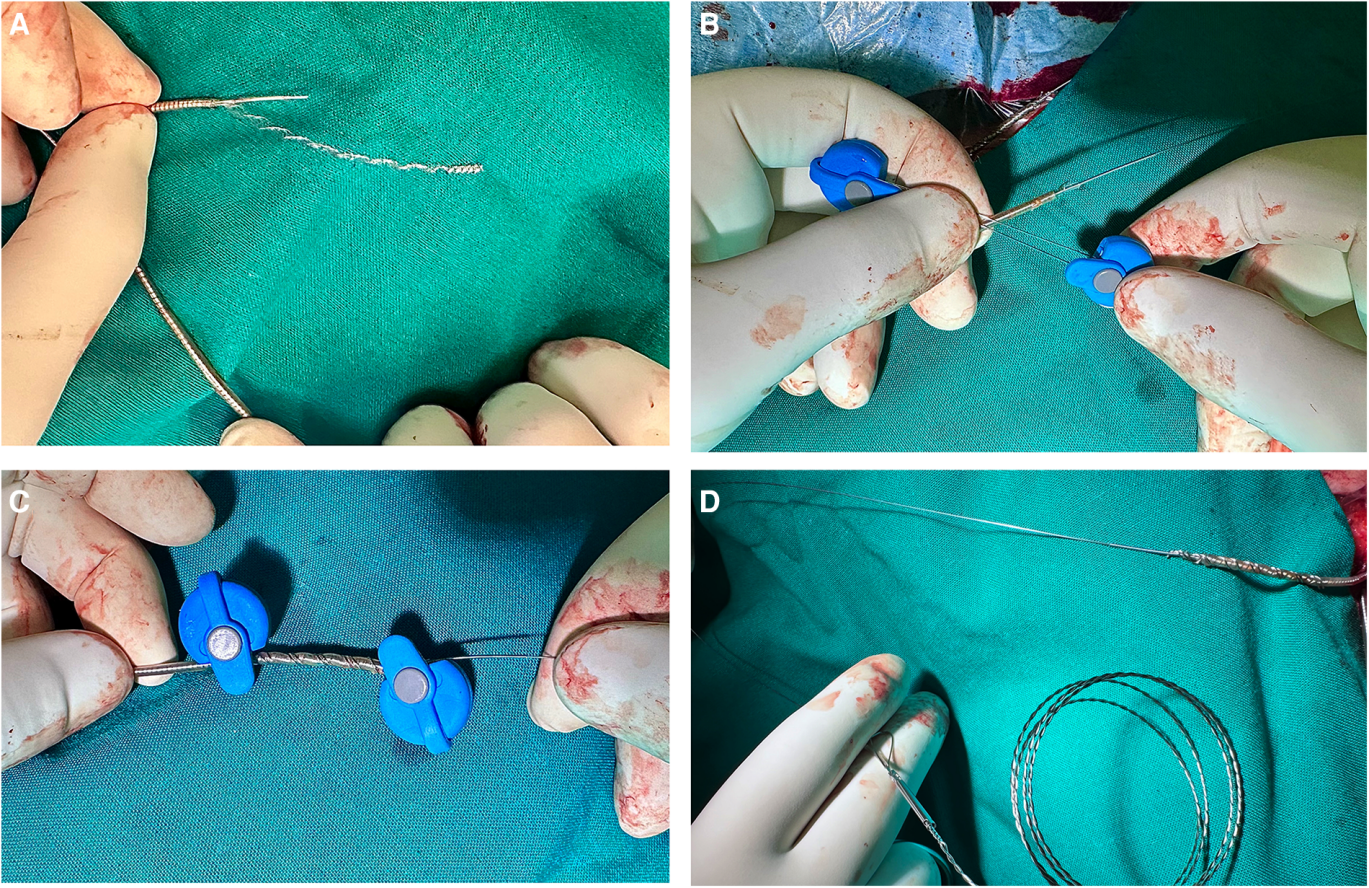
Preparing the lead for extraction. **(A)** The lead connector and the outer coating of the lead are cut off to expose the inner lumen. **(B)** The Liberator stylet is advanced to the tip of the lead through the lead lumen. **(C, D)** A One-Tie compression coil is wound around the lead to bind the Liberator stylet to the body of the lead.

The lead extraction was carried out using the subclavian approach. After removing the sutured sleeve and adhesions, extraction was initially attempted through simple manual traction. No resistance was encountered in the superior vena cava but unfortunately the SD lead was densely adhered in the interventricular septum, necessitating the use of traction devices to facilitate the extraction. The proximal pin of the electrode was resected, and a Liberator locking stylet (Cook Medical, Bloomington, IN, USA) was advanced to the tip of the electrode to provide internal support. To ensure secure binding to the locking stylet and the proximal components of the lead, a One-Tie Compression Coil (Cook Medical) was wound around the proximal lead end (Figure 1). With controlled traction, the electrode was completely extracted.

**Figure 2:**
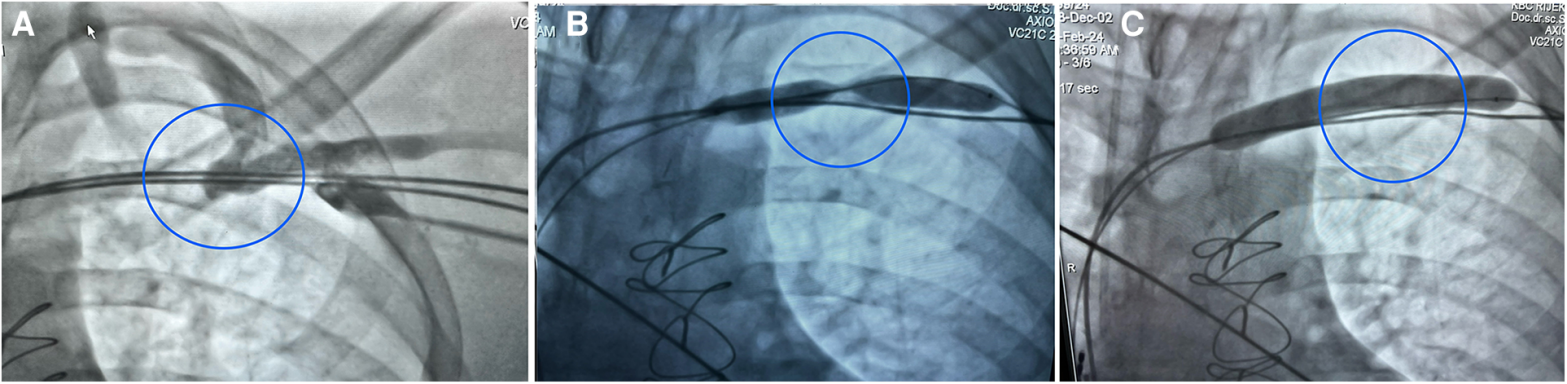
Intraprocedural fluoroscopy images. **(A)** Complete distal left subclavian stenosis with collateral drainage to the internal jugular vein. **(B)** Venoplasty carried out after lead extraction with an Armada peripheral balloon passed along a guidewire over the stenotic segment. **(C)** A completely inflated Armada peripheral balloon.

After the extraction, a new lead implantation was pursued by puncturing the axillary vein, but the aforementioned vein occlusion prevented the hydrophilic guidewire from passing through. The occlusion was traversed using a Gaia Third (Asahi Intecc, Aichi, Japan) guidewire inserted into a hydrophilic KA2 catheter (6F, Merit Medical, South Jordan, UT, USA). The operator then performed a subclavian venoplasty with a balloon for peripheral artery stenosis dilatation (Armada 10.0 mm × 80 mm, 10 atm; Abbot Vascular, Abbott, Santa Clara, CA, USA) (Figures 2B,C). The arterial sheath was then exchanged for a venous one (7F, Biotronik LI-7), and a C315HIS catheter (Medtronic Inc.) was inserted.

**Figure 3:**
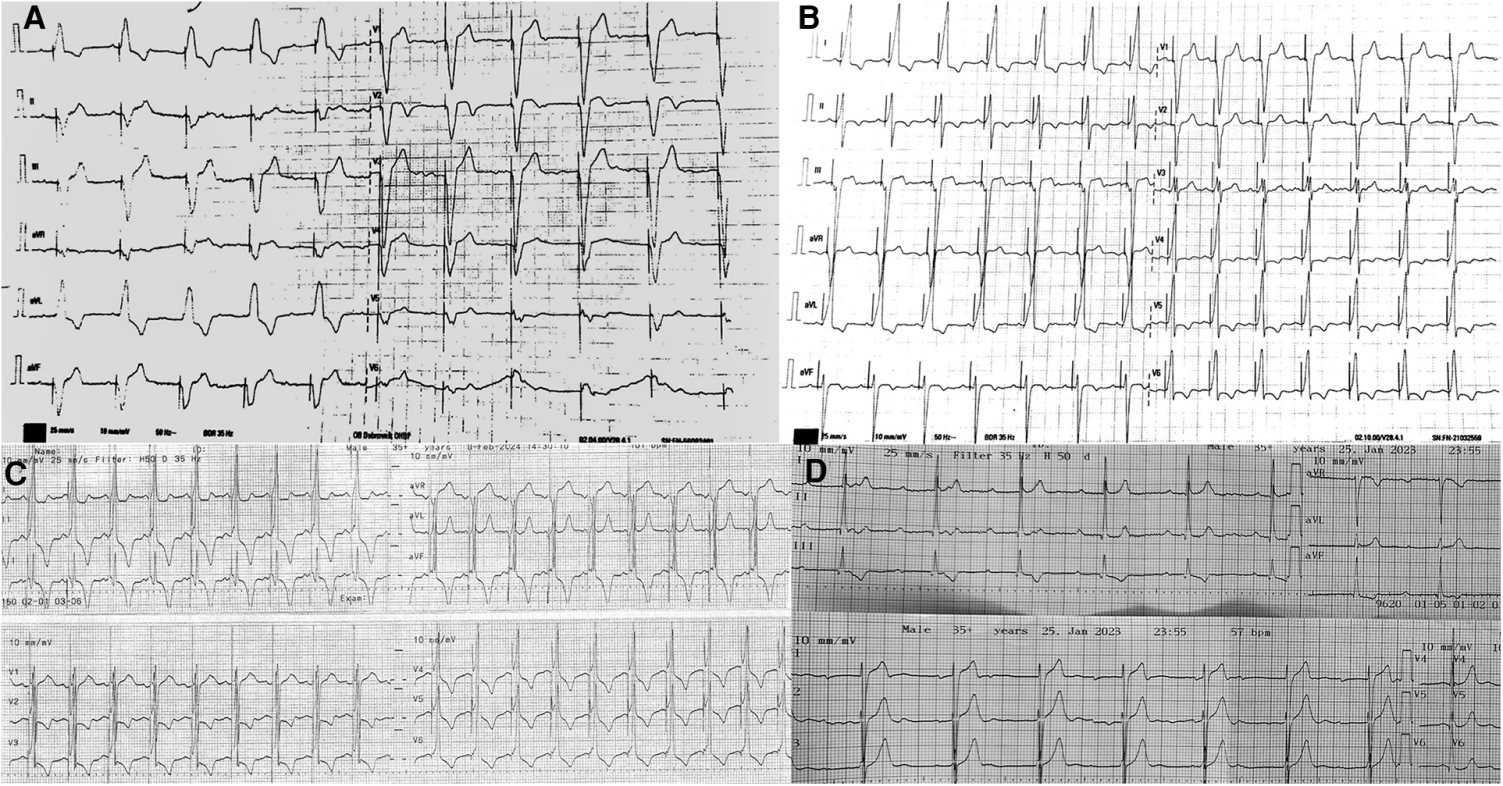
Twelve lead ECG. **(A)** Ventricular stimulation from a single-chamber pacemaker with atrial sensing (VDD). Atrioventricular synchrony is lost due to atrial undersensing. **(B)** Left bundle branch area pacing with typical positive lead II+and negative lead III deflection. The QRS duration is 120 ms; the LVAT in V6 is 100 ms; and the V6-V1 interpeak interval is <33 ms—changes due to lead microdislocation. **(C)** Significant QRS width reduction after implantation at the area of the His bundle. With a unipolar pacing output of 2.5 mV at 1.0 ms, QRS is 90 ms as a result of non-selective His bundle capture. **(D)** Non-stimulated native ECG recorded after extraction with the QRS duration of 85 ms indicating intact intrinsic conduction after extraction.

**Figure 4:**
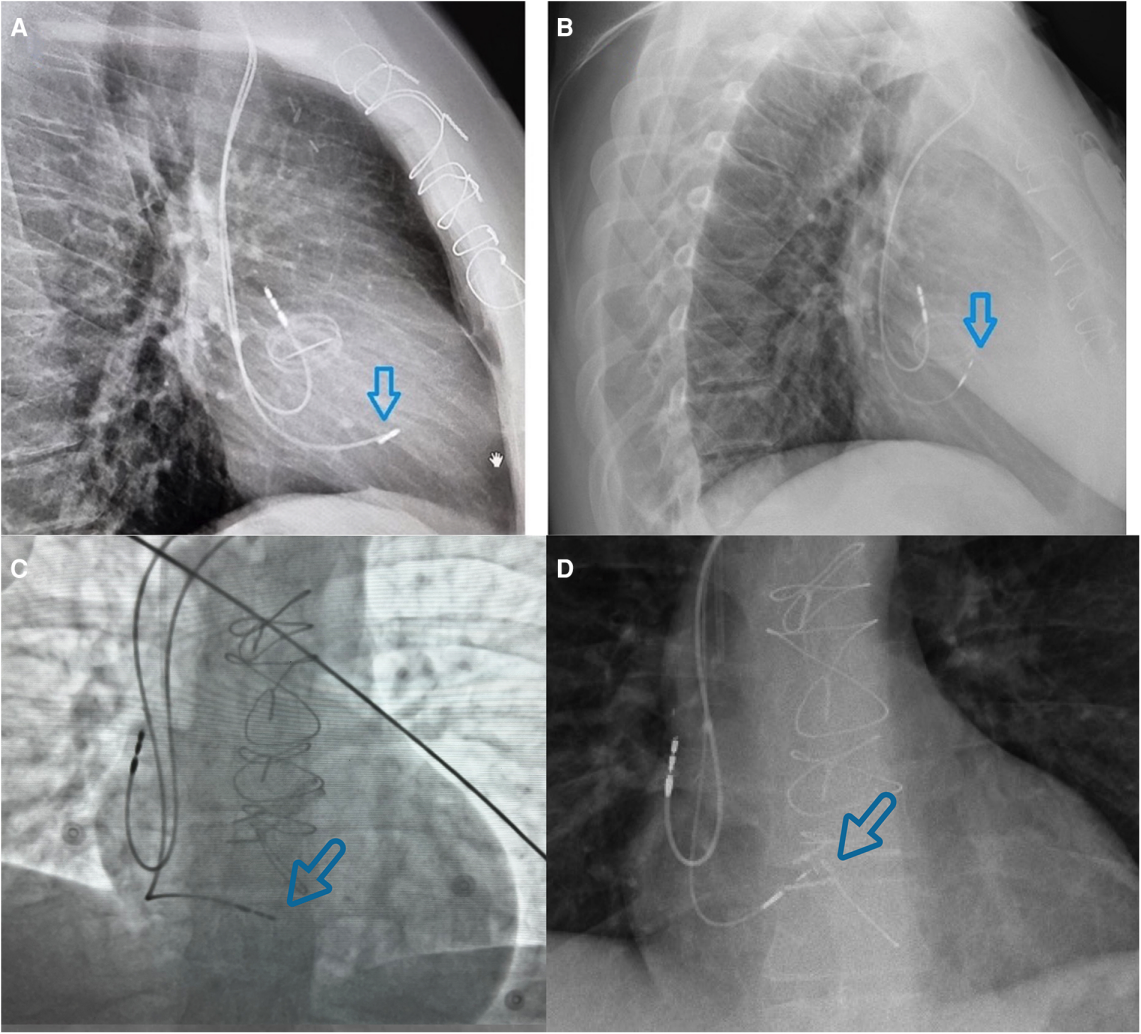
Different views to compare lead position in left bundle branch area pacing (left images) and at the area of the His bundle (right images). **(A)** Lateral view chest x-ray image of the ventricular lead positioned in the LBBAP area. **(B)** Lateral view chest x-ray image of the ventricular lead in the area of the His bundle (thinner lumenless lead; approximately 2 cm higher than the LBBAP lead). **(C)** Fluoroscopic antero-posterior view of the ventricular lead positioned in the LBBAP area. **(D)** Antero-posterior x-ray view of the ventricular lead in the area of the His bundle.

LBBAP was attempted using a SelectSecure 3830 lead (69 cm) (Medtronic Inc.). Despite multiple attempts at different positions, septal fibrosis prevented the electrode from being inserted at the site of optimal QRS morphology. Consequently, the lead was placed at the distal His position, resulting in non-selective His capture QRS morphology and a greater narrowing of QRS duration (QRS duration at His position, 90 ms) compared with the previous non-optimal LBBAP (QRS duration at LBBAP, 120 ms) (Figures 3B,C). The parameters were a selective His capture threshold of 1.8 A at 1.0 ms, ventricular threshold of 0.7 A at 1.0 ms, and sensing of 2.5 mV. The leads were connected to a permanent pacemaker with an extended battery life (Attesta LDR, MRI SureScan, Medtronic Inc.) (Figure 4).

The total procedural time was 130 min, with a fluoroscopy time of 49 min, radiation dose of 851 mGy, and dose area product of 4,618 µGy m^2^. The postoperative course was uneventful, and the patient was discharged the following day.

## Discussion

We presented a case of a deep septal SD lead extraction using traction tools, 27 months after implantation, without complications, demonstrating the feasibility of SD lead extraction in long-term settings. An increasing number of complex septal extractions can be expected in the future, and our report aims to provide valuable guidance on managing such cases. To the best of our knowledge, this is the first case of a successful long-dwelling LBBAP SD lead extraction using traction tools.

Our case demonstrates the feasibility of lead removal from the deep septal left bundle branch location when simple manual traction is insufficient. Monitoring the entire procedure with intracardiac echocardiography reduced radiation exposure and provided safer guidance and showed no evidence of ventricular septal defect and no tricuspid regurgitation at the end of the procedure. Adhesions of the lead tip were so tight that they could not be removed with simple manual traction. The stiffness of the lead tip adhesions was likely due to the extended lead-dwelling time, which was much longer than in any other reported cases (8–10). In addition, the SD lead (Solia) is thicker than the SelectSecure lead (5.9F compared with 4.1F), creating more adhesions between the lead and the adjacent myocardium.

To date, there have been only two case reports of successful complete percutaneous extraction of an SD lead from the deep septum, and our case differs to those in few ways. First, in the other cases, the lead dwell time was relatively short (4 and 10 months after implantation), and second, both were extracted through manual traction using standard non-locking stylets (8, 9). Longer dwell leads are likely to present significantly more challenges related to fibrosis and calcification and will probably require mechanical tools for extraction, which aligns with our case. Native ECG recorded after implantation showed a narrow QRS complex, proving that the patient's conduction system was not damaged during the extraction (Figure 3D).

To reinforce the lead and reduce the risk of lead disruption, the lead was prepared with a locking stylet and compression coil. We chose the Liberator stylet because it focuses the locking mechanism strength at the distal tip of the stylet, providing focal traction at the tip of the lead. In contrast, other available locking stylets, such as the Spectranetics LLD (Spectranetics, Colorado Springs, CO, USA), grab the complete lead except for the first few centimetres, making it less ideal in this case. Based on the reported literature and the operator's experience, we did not retract the active fixation screw to prevent helix fracture (8). It is also important to highlight that we avoided any rotating manoeuvres during the traction. Counterclockwise rotation manoeuvres were not attempted due to the fear of tip fracture consequent to screw entanglement. Ruptures of the fixating mechanism have been reported in several cases (8, 10), and are presumably the consequence of the screwing mechanism's fragility.

Data on transvenous lead extraction in LBBAP are limited to retrospective data sets and case reports, which mostly describe the extraction of lumenless (LL) leads, with the main concern being the lack of a lumen for placing a locking stylet for complex extractions (11). In addition, the number of SD leads for LBBAP implants is increasing (7, 12) and, as such, concerns about long-term extractability are growing.

A progressive decline in left ventricular function was noted due to septal dyskinesia, despite guideline-directed medical therapy. We speculate that only a partial capture of the anterior fascicle was achieved, or only the left ventricular deep septal pacing was obtained, and over time, the QRS widened, and pacing parameters were less optimal due to microdislocation. The most probable cause for lead microdislocation is a fibrotic interventricular septum developed following aortic valve replacement. This argument also explains our unsuccessful LBBAP lead deployment, as septal scar is associated with higher implant failure rates (13). With the achieved narrow QRS and synchronised left ventricular activity, recovery of left ventricular function is anticipated. We achieved a satisfying threshold in the distal His area.

Our patient was discharged the day after the procedure, but in the past year, three studies have confirmed the safety of the “same-day discharge” approach following transvenous lead extractions (14–16). The most recent study demonstrated non-inferior outcomes for patients who were discharged on the same day compared with those who were not, supporting the concept of lead extraction feasibility (14).

In conclusion, we report a complex case of long-dwelling SD lead extraction used for LBBAP, followed by His lead implantation after venoplasty due to ipsilateral subclavian vein occlusion. As discussed, an increasing number of complex extractions can be expected in the future, demanding a range of skills from operators. This case provides valuable guidance on managing more complex cases until new tools and techniques are developed.

## Data Availability

The original contributions presented in the study are included in the article/Supplementary Material, further inquiries can be directed to the corresponding author.

## References

[1] Marcial JM Worley SJ. Venous system interventions for device implantation. Card Electrophysiol Clin. (2018) 10(1):163–77. 10.1016/j.ccep.2017.11.017 29428138

[2] Abdelrahman M Subzposh FA Beer D Durr B Naperkowski A Sun H Clinical outcomes of His bundle pacing compared to right ventricular pacing. J Am Coll Cardiol. (2018) 71:2319–30. 10.1016/j.jacc.2018.02.048 29535066

[3] Sharma PS Patel NR Ravi V Zalavadia DV Dommaraju S Garg V Clinical outcomes of left bundle branch area pacing compared to right ventricular pacing: results from the Geisinger-Rush conduction system pacing registry. Heart Rhythm. (2022) 19:3–11. 10.1016/j.hrthm.2021.08.033 34481985

[4] Vijayaraman P Sharma PS Cano Ó Ponnusamy SS Herweg B Zanon F Comparison of left bundle-branch area pacing to biventricular pacing in candidates for resynchronization therapy. J Am Coll Cardiol. (2023) 82(3):228–41. 10.1016/j.jacc.2023.05.006 37220862

[5] Zanon F Ellenbogen KA Dandamudi G Sharma PS Huang W Lustgarten DL Permanent His-bundle pacing: a systematic literature review and meta-analysis. Europace. (2018) 20(11):1819–26. 10.1093/europace/euy058 29701822

[6] Sharma PS Vijayaraman P. Conduction system pacing for cardiac resynchronisation. Arrhythm Electrophysiol Rev. (2021) 10(1):51–8. 10.15420/aer.2020.45 33936744 PMC8076975

[7] De Pooter J. My preferred approach to left bundle branch area pacing: stylet-driven leads. Heart Rhythm O2. (2022) 4(2):154–6. 10.1016/j.hroo.2022.12.011 36873317 PMC9975020

[8] Agudo CA Jaén EG Sánchez DJ Urda VC Ramos JT Lozano IF. Extraction of a fractured pacemaker lead in the left bundle branch area using a snare via a femoral approach. J Interv Card Electrophysiol. (2023) 66(2):239–40. 10.1007/s10840-022-01325-7 35951215

[9] Bonfanti P Mantovani A Refugjati T Sormani L Corrado G. Extraction of stylet-driven pacing lead for left bundle branch area pacing. J Arrhythm. (2023) 40(1):174–6. 10.1002/joa3.12957 38333388 PMC10848579

[10] le Polain de Waroux JB Wielandts JY Gillis K Hilfiker G Sorgente A Capulzini L Repositioning and extraction of stylet-driven pacing leads with extendable helix used for left bundle branch area pacing. J Cardiovasc Electrophysiol. (2021) 32(5):1464–6. 10.1111/jce.15030 33825263

[11] Wijesuriya N Elliott MK Mehta V Behar JM Niederer S Wilkoff BL Transvenous lead extraction in conduction system pacing. Front Physiol. (2022) 13:993604. 10.3389/fphys.2022.993604 36035491 PMC9410714

[12] Zanon F Marcantoni L Pastore G Baracca E. Left bundle branch pacing by standard stylet-driven lead: preliminary experience of two case reports. Heart Rhythm Case Reports. (2020) 6:614–7. 10.1016/j.hrcr.2020.06.005 32983878 PMC7498513

[13] Ali N Arnold AD Miyazawa AA Keene D Peters NS Kanagaratnam P Septal scar as a barrier to left bundle branch area pacing. Pacing Clin Electrophysiol. (2023) 46(9):1077–84. 10.1111/pace.14804 37594233

[14] Atteya G Alston M Sweat A Saleh M Beldner S Mitra R Same-day discharge after transvenous lead extraction: feasibility and outcomes. Europace. (2023) 25(2):586–90. 10.1093/europace/euac185 36575941 PMC9934987

[15] Dagher L Tfaily MA Vavuranakis M Bhatia NK Westerman SB Shah AD Safety of same-day discharge after lead extraction procedures. Heart Rhythm. (2023) 20(12):1669–73. 10.1016/j.hrthm.2023.08.010 37591366

[16] Gianni C Elchouemi M Helmy R Spinetta L La Fazia VM Pierucci N Safety and feasibility of same-day discharge following uncomplicated transvenous lead extraction. J Cardiovasc Electrophysiol. (2024) 35(2):278–87. 10.1111/jce.16147 38073051

