# Supplementary figures and images for: Case Report: Extraction of a stylet-driven lead for left bundle branch area pacing >2 years after implantation
Source: Front Cardiovasc Med. 2024 Aug 26;11:1457025. doi: 10.3389/fcvm.2024.1457025 (PMC11381278; doi:10.3389/fcvm.2024.1457025)

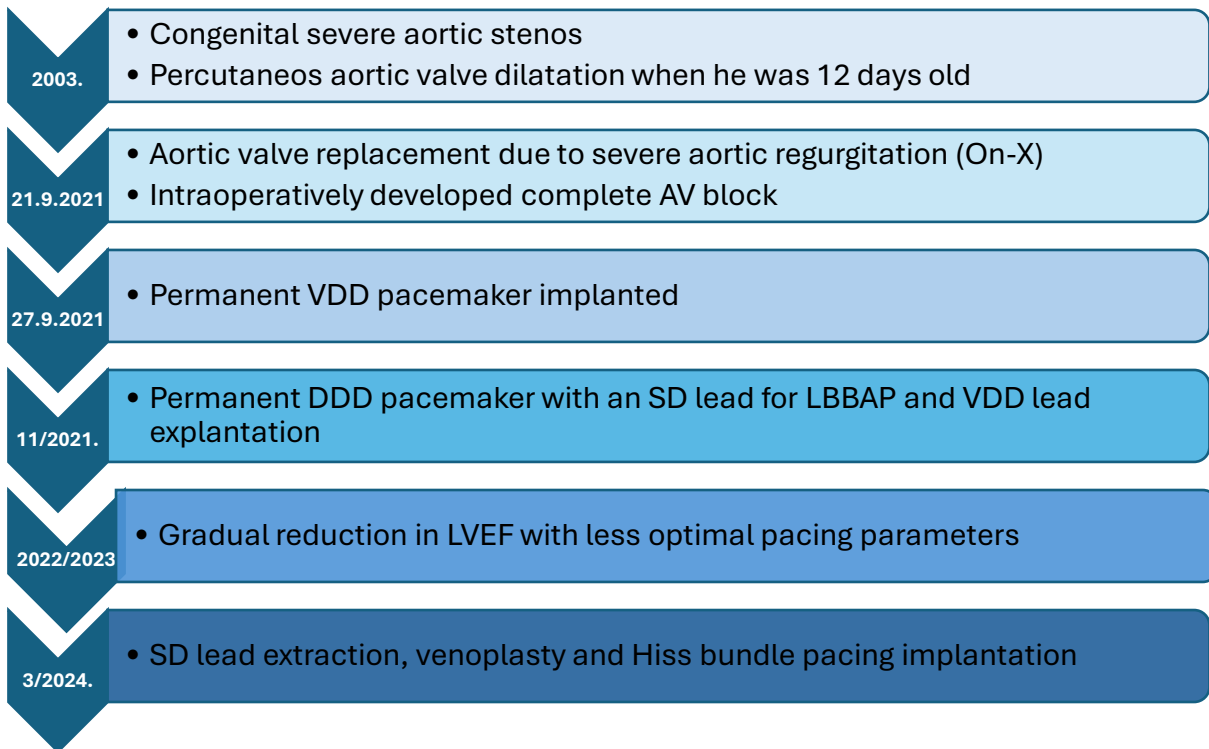

Supplement: Supplementary file 1 [file Datasheet1.pdf]
